# Employing digital PCR for enhanced detection of perinatal *Toxoplasma gondii* infection: A cross-sectional surveillance and maternal-infant outcomes study in El Salvador

**DOI:** 10.1371/journal.pntd.0012153

**Published:** 2024-05-20

**Authors:** Mary K. Lynn, Marvin Stanley Rodriguez Aquino, Pamela Michelle Cornejo Rivas, Xiomara Miranda, David F. Torres-Romero, Hanson Cowan, Madeleine M. Meyer, Willber D. Castro-Godoy, Mufaro Kanyangarara, Stella C. W. Self, Berry A. Campbell, Melissa S. Nolan

**Affiliations:** 1 Department of Epidemiology and Biostatistics, Arnold School of Public Health, University of South Carolina, Columbia, South Carolina, United States of America; 2 Health Research and Development Center (CENSALUD), University of El Salvador, San Salvador, El Salvador; 3 Hospital Nacional “Dr Jorge Mazzini Villacorta”, Ministerio de Salud, Sonsonate, El Salvador; 4 Department of Chemistry and Pharmacy, University of El Salvador, San Salvador, El Salvador; 5 Department of Obstetrics and Gynecology, Prisma Health, Columbia, South Carolina, United States of America; Aberystwyth University - Penglais Campus: Aberystwyth University, UNITED KINGDOM

## Abstract

*Toxoplasma gondii* is a parasitic infection that can be transmitted in utero, resulting in fetal chorioretinitis and other long-term neurological outcomes. If diagnosed early, pregnancy-safe chemotherapeutics can prevent vertical transmission. Unfortunately, diagnosis of acute, primary infection among pregnant women remains neglected, particularly in low-and-middle-income countries. Clinically actionable diagnosis is complex due to the commonality of infection during childhood and early adulthood which spawn long-last antibody titers and historically unreliable direct molecular diagnostics. The current study employed a cross-sectional *T*. *gondii* perinatal surveillance study using digital PCR, a next generation molecular diagnostic platform, and a maternal-fetal outcomes survey to ascertain the risk of vertical toxoplasmosis transmission in the Western Region of El Salvador. Of 198 enrolled mothers at the time of childbirth, 6.6% had evidence of recent *T*. *gondii* infection—85% of these cases were identified using digital PCR. Neonates born to these acutely infected mothers were significantly more likely to meconium aspiration syndrome and mothers were more likely to experience labor and delivery complications. Multivariable logistic regression found higher maternal *T*. *gondii* infection odds were associated with the presence of pet cats, the definitive *T*. *gondii* host. In closing, this study provides evidence of maternal *T*. *gondii* infection, vertical transmission and deleterious fetal outcomes in a vulnerable population near the El Salvador-Guatemala border. Further, this is the first published study to show clinical utility potential of digital PCR for accurate diagnosis of congenital toxoplasmosis cases.

## Author summary

Toxoplasmosis infection is globally prevalent but can cause severe negative neonatal health outcomes when acquired congenitally. Transmission from mother to neonate depends upon a mother’s first-time infection and the stage of pregnancy when mother is infected. The causative agent of toxoplasmosis is a protozoan parasite that contributes to the common difficulties in identifying a mother’s primary acute infection. Commonly, serologic testing is administered to identify antibodies to the infection, along with avidity testing that helps to determine current versus past infection. Digital PCR is a next-generation molecular tool that can identify very small quantities of pathogens. This tool was utilized in addition to typical serology plus avidity testing to identify suspected primary *T*. *gondii* infections in banked blood and serum samples from Salvadoran women at the time of labor and delivery. Health outcomes surveys were also utilized from the original study to determine associations between recent *T*. *gondii* infections during pregnancy and maternal-neonatal pregnancy and birth outcomes. We identified 13 women with evidence of recent *T*. *gondii* infection during pregnancy, 11 of which were identified through dPCR. Maternal infection was associated with negative maternal outcomes including pregnancy complications, and associations were found between infection and neonatal meconium aspiration syndrome. Maternal infection was also associated with owning pet cats. This study utilized a new molecular tool to aid in identification of T. gondii primary infection, a neglected disease that is historically difficult to identify through traditional methods. Digital PCR could be useful for toxoplasmosis testing in future studies and clinical settings.

## Introduction

*Toxoplasma gondii* is a highly diverse apicomplexan protozoan responsible for the zoonotic infection toxoplasmosis [1–3]. This infection is prevalent globally, and over one third of the world’s population is thought to be infected, with the majority of infections presenting asymptomatically [1,4–7]. Transmission occurs when humans ingest tissue cysts from undercooked meat or sporulated oocysts from the environment. Oocysts are spread through the environment via the feces of the definitive feline host species and may contaminate food or water [2]. When chronically infected, cysts remain within brain and muscle tissues for the lifetime of the host [3]. In low and middle income countries with consistently warm temperatures, the risk of toxoplasmosis infection may be higher as oocysts can persist for longer periods within the environment [2]. The infection circulates among a wide range of mammalian hosts and can also be transmitted vertically through the placenta [7].

Human congenital *T*. *gondii* transmission is a major global health issue, attributable to a worldwide burden of 1.2 million disability-adjusted life years (DALYS) [5]. The foremost concern for congenital disease is primary, acute infection during pregnancy—where the woman’s first lifetime infection occurs during her pregnancy [8]. Immunocompromised women may also transmit the parasite across the placenta with a reactivated infection [8]. Infection within earlier stages of pregnancy is associated with more severe symptoms and disease sequelae later in the child’s life [5,7,9]. Conversely, the risk of vertical transmission increases the further along in pregnancy in which *T*. *gondii* is primarily acquired, with the highest risk related to maternal seroconversion between 10 and 24 weeks of gestational age [9].

Though not well-defined, the mechanism of vertical transmission has been associated with specific fetal and maternal immune responses to the parasite and inflammatory response derived placental damage [1]. Clinical outcomes of congenital transmission can be severe, impacting the central nervous system, resulting in stillbirth, neonatal death, encephalitis, chorioretinitis, and intracranial calcifications [2,4,10]. Other commonly reported symptoms from clinical studies include microcephaly, seizures, hepatosplenomegaly, thrombocytopenia, rash, jaundice and anemia [4,5]. Classically indicative hallmarks of congenital toxoplasmosis include intracranial calcifications, chorioretinitis, and hydrocephalus. However in practice, this triad of symptoms only presents in a small number of cases, with hydrocephalus occurring in approximately 4% of vertically infected, symptomatic neonates [9]. The most common disease presentations are related to ocular infection and vision difficulties [9]. Most congenital infections will show no symptoms at the time of birth; however, 30% will nonetheless develop lasting symptoms of ocular disease by age 12 [9].

Global seroprevalence among pregnant women is approximately 33% and 1% for IgG and IgM, respectively, though significant variation exists by world region and national income [5,9,10]. Diagnostic challenges exist for *T*. *gondii* particularly surrounding temporality of infection [11,12]. *T*. *gondii* specific IgM may react to protozoal antigens in the absence of infection and may persist for months or years [12]. IgG avidity tests help to distinguish recent from past infections, as antigen-antibody binding strength increases over time after primary infection. Molecular methods have also been used to determine active infection to assist in clarifying acute infections, due to the complexity of assessing serologic test results [12].

A paucity of research exists regarding *T*. *gondii* maternal or congenital infection in Central America. Meta-analysis estimates suggest national IgG seroprevalence in El Salvador is nearly 52% and IgM seroprevalence is approximately 0.9%[10]. Prenatal screening guidelines from the El Salvador Ministry of Health recommend all women be tested for HIV and syphilis during prenatal care visits [13]: toxoplasmosis is only tested for in patients with consistent clinical symptoms. Therefore, few maternal toxoplasmosis epidemiology and transmission risk studies have been conducted in El Salvador, and the majority are limited to the gray literature. These studies demonstrate between 17–27% IgG seroprevalence and up to 15% IgM seroprevalence among women of childbearing age [14,15]. The incidence of neonatal infection is also not well described. However, fatal congenital toxoplasmosis has been recently reported in the eastern department of Usulután [16]. Studies of microcephalic infants and low birth-weight neonates presenting to a national hospital reported 7% and 1% seropositivity for *T*. *gondii* respectively [17,18]. This study aimed to assess the prevalence of active *T*. *gondii* infection and associated maternal and neonatal outcomes among women presenting for childbirth at the referent public hospital in Sonsonate, El Salvador.

## Methods

### Ethics statement

The use of banked sera samples for *Toxoplasma gondii* testing was approved by the National Ethics Committee of El Salvador (IRB N° 0005660). Adult participants provided written informed consent. For minors of age <18 years, both written informed participant assent and parental/guardian consent were obtained for participation.

### Participant recruitment

From March to September 2022, 198 patients ≥15 years presenting for labor and delivery at Hospital Nacional General “Dr. Jorge Mazzini Villacorta” (Hospital Mazzini), the referent public hospital in Sonsonate, El Salvador, agreed to participate in the study. Following consent, participants donated blood samples for infectious disease testing, as well as a small urine sample to detect indicators of possible pre-eclampsia. Maternal sera for these 198 participants were available for *T*. *gondii* serology testing and their labor and delivery health records were available for clinical-epidemiologic analysis.

### Serologic and Molecular Methods

Banked serum samples were screened for the presence of IgG and IgM antibodies against *T*. *gondii* by enzyme-linked immunosorbent assay (ELISA) (Calbiotech, El Cajon, CA, USA). IgG avidity testing was further performed to distinguish past versus recent infection using Avidity *Toxoplasma gondii* IgG ELISA kit (Abcam, Waltham, MA, USA). To confirm infection, digital polymerase chain reaction (dPCR) testing was performed following DNA extraction from whole blood samples mixed with DNA/RNA Shield preservative using QIAamp 96 Virus QIAcube HT Kit for DNA and RNA (Qiagen, Germantown, MD, USA). Presence of parasite DNA was determined using a next-generation digital polymerase chain reaction (dPCR) assay on the QIAcuity Four Digital PCR system (Qiagen, Germantown, MD) and Qiagen 24-well 26k Nanoplates (Qiagen, Germantown, MD). Previously validated primers and probes targeting REP529, the highly conserved and repetitive 529 bp fragment of the parasite genome were used [19–21]. Reaction mix was prepared using Qiagen QIAcuity Probe PCR kit (Qiagen, Germantown, MD), AluI cutter restriction enzyme (New England Biosystems, Ipswich, MA) and primers and FAM probe from Integrated DNA Technologies (IDT, Coralville, IA). Genomic DNA from *Toxoplasma gondii* strain RH (ATCC 50174D) was purchased from American Type Culture Collection (Manassas, VA USA) for use as a positive control. Digital PCR Partition classification was conducted via Umbrella in RStudio, R version 4.3.0 using a hard thresholding value of p_0,A_ ≥ 0.95 and nuclease free water non-template controls [22]. The following reaction mixture prepared: 10 uL Qiagen master mix,4 uL 10 mM forward/reverse primers, 2 uL 10 mM FAM probe, 1 uL AlUI restriction enzyme, 33 uL nuclease free water, and 5 uL template DNA. Gradient PCR was conducted to determine the following optimal cycle conditions: 95.0° for 5 min then 50 cycles of 95.0° for 30 seconds, 56.0° for 1 min, and 72.0° for 30 seconds. A 1:10 serial dilution (1 – 1x10^-6^ μg/μL) of positive standards was used to determine the method detection limit for our system per the method described by Hunter et al [23]. The specific dPCR target sequences are listed in S1 Table. The following formula was then used to assess the limit of detection:

$$\frac{copies}{\mu L}=\frac{X\frac{ng}{\mu L}*6.022E23\frac{copies}{mol}}{8E7bp*660\frac{g}{mol*bp}*1E9\frac{ng}{g}}$$

**Fig 1:**
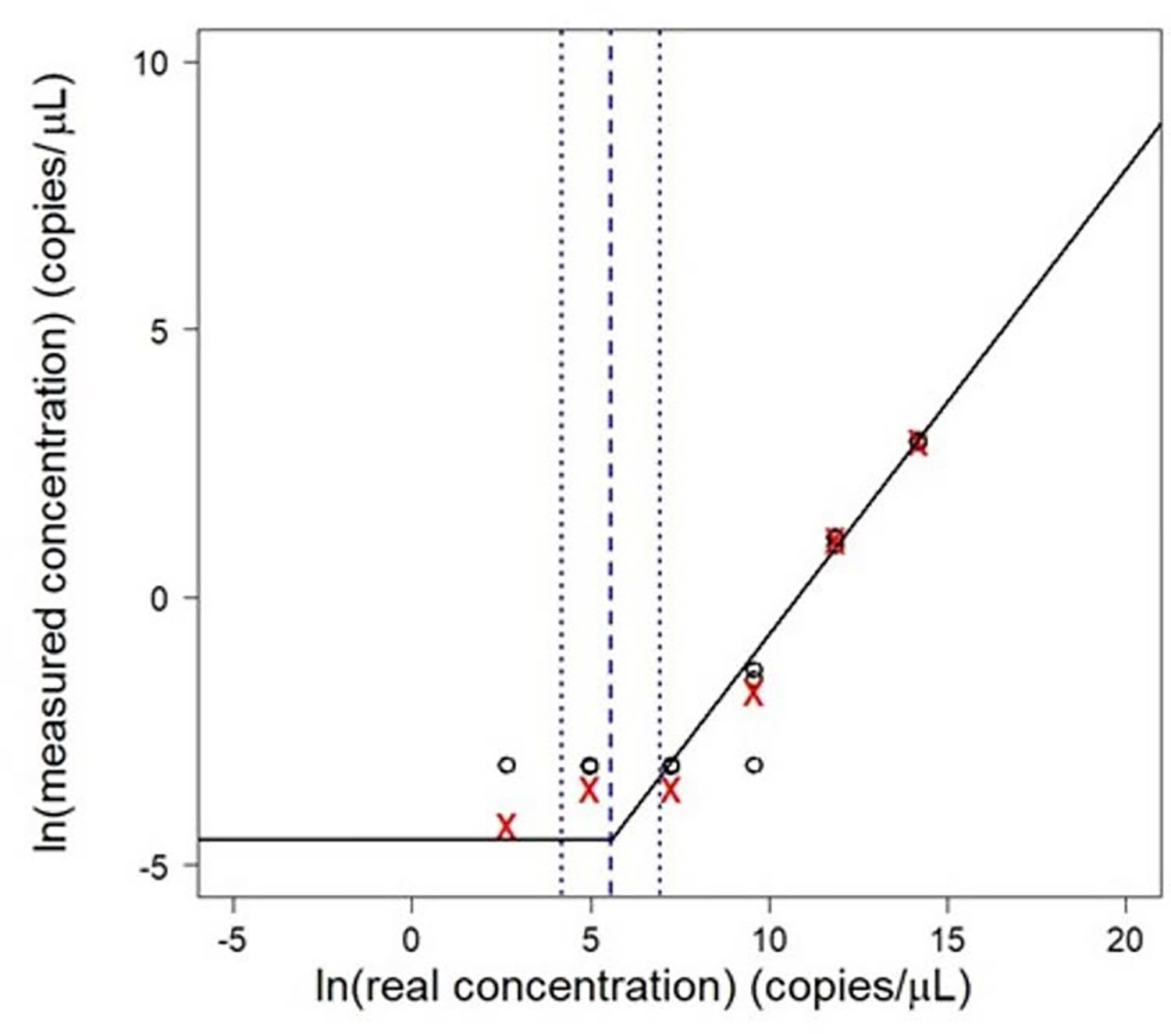
Calculation results for the limit of detection. The final limit of detection for the dPCR reaction was determined to be 0.011 copes per uL.

Results of these calculations are shown in Fig 1, finding a final limit of detection to be 0.011 copies per uL.

**Fig 2:**
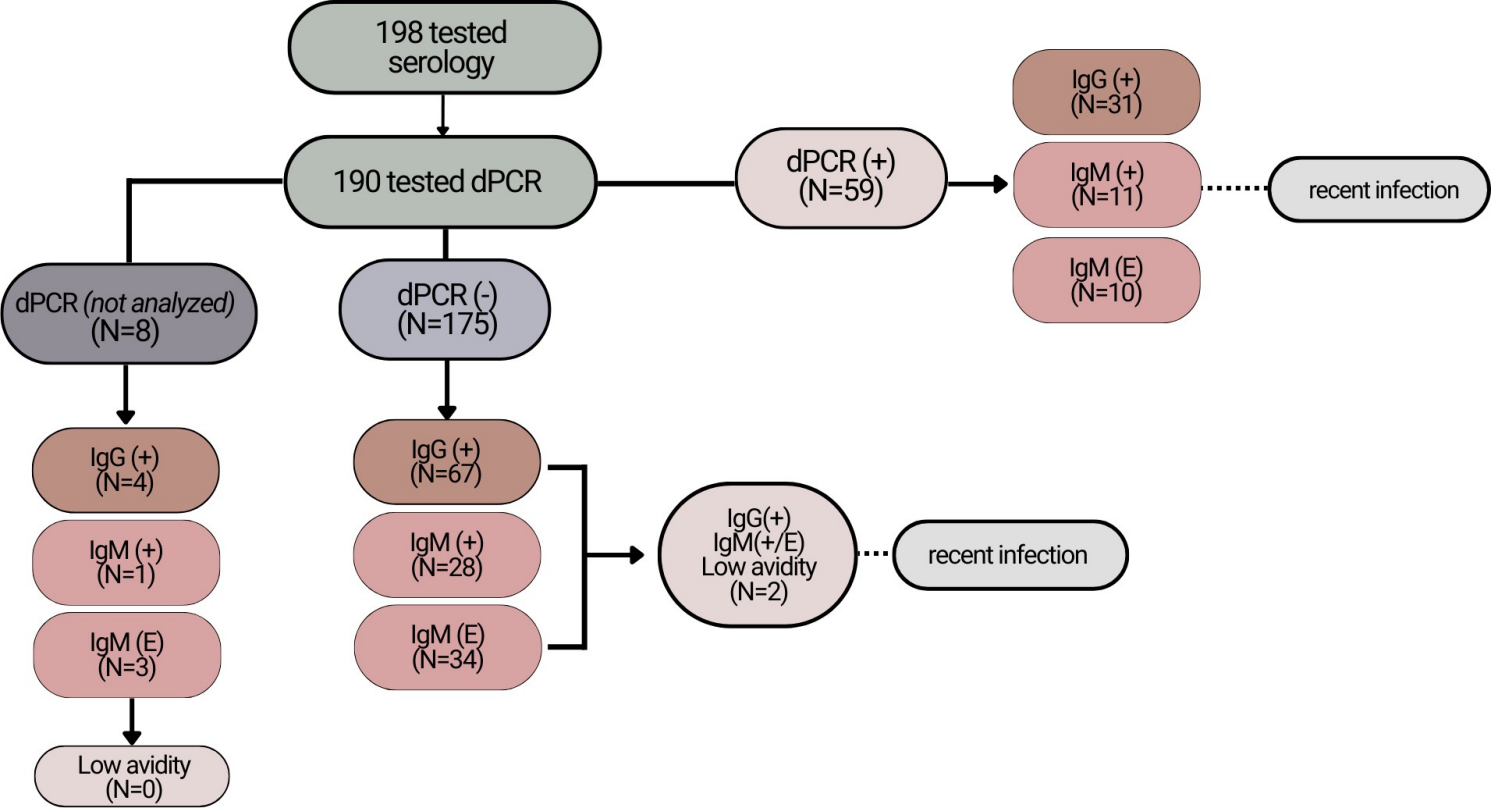
Summary of testing algorithm and classification of recent *T*. *gondii* infection within study population. Among Salvadoran perinatal women at the time of parturition, 17 individuals had evidence of recent *T*. *gondii* infection within the last 16 weeks. Due to differing quantities of banked sera and whole blood, some individuals’ samples did not receive dPCR testing and results relied on previously published clinical diagnostic algorithms of serology and avidity testing.

All samples underwent serology testing; however, not all samples underwent dPCR or avidity testing in cases of insufficient remaining biological samples. The number of banked samples that underwent each testing platform is described in Fig 2.

### Interpretation of results

Participant samples were classified as having evidence of recent infection, defined as infection within the last 16 weeks, through assessing the combination of dPCR, IgG and IgM serology, and IgG avidity testing results as previously described [24–26]. Due to the complexity of elucidating infection timing in endemic settings, this study relied on the previously published infectious disease guidelines for interpretations of positive (+), negative (-) and equivocal (E) serology and avidity test results [24]. All banked whole blood samples with sufficient volume after DNA extraction underwent digital PCR (N = 190). Digital PCR was then used in combination with serologic results to determine evidence of recent infections. Specifically, recently infected mothers in this study were defined as meeting at least one of the following criteria:

- Sera sample determined IgG positive AND IgM result either positive or equivocal AND low avidity testing result.
- Sera sample determined IgM positive AND whole blood sample detected parasite DNA via dPCR

A simplified flow diagram of our testing and interpretation algorithm is presented in Fig 2 for clarity.

### Statistical and spatial analysis

Fisher’s exact test was used to detect univariate associations between recent *T*. *gondii* infection, and 1) maternal and neonatal health outcomes abstracted from clinical charts, and 2) risk factors for infection obtained from the participant questionnaires. Multivariable logistic regression was used to determine statistically significant odds ratios to describe the relationship between recent toxoplasmosis infection and risk factors for infection. All statistical analyses were performed in RStudio version 4.1.1 (RStudio, PBC, Boston, MA).

## Results

**Fig 3:**
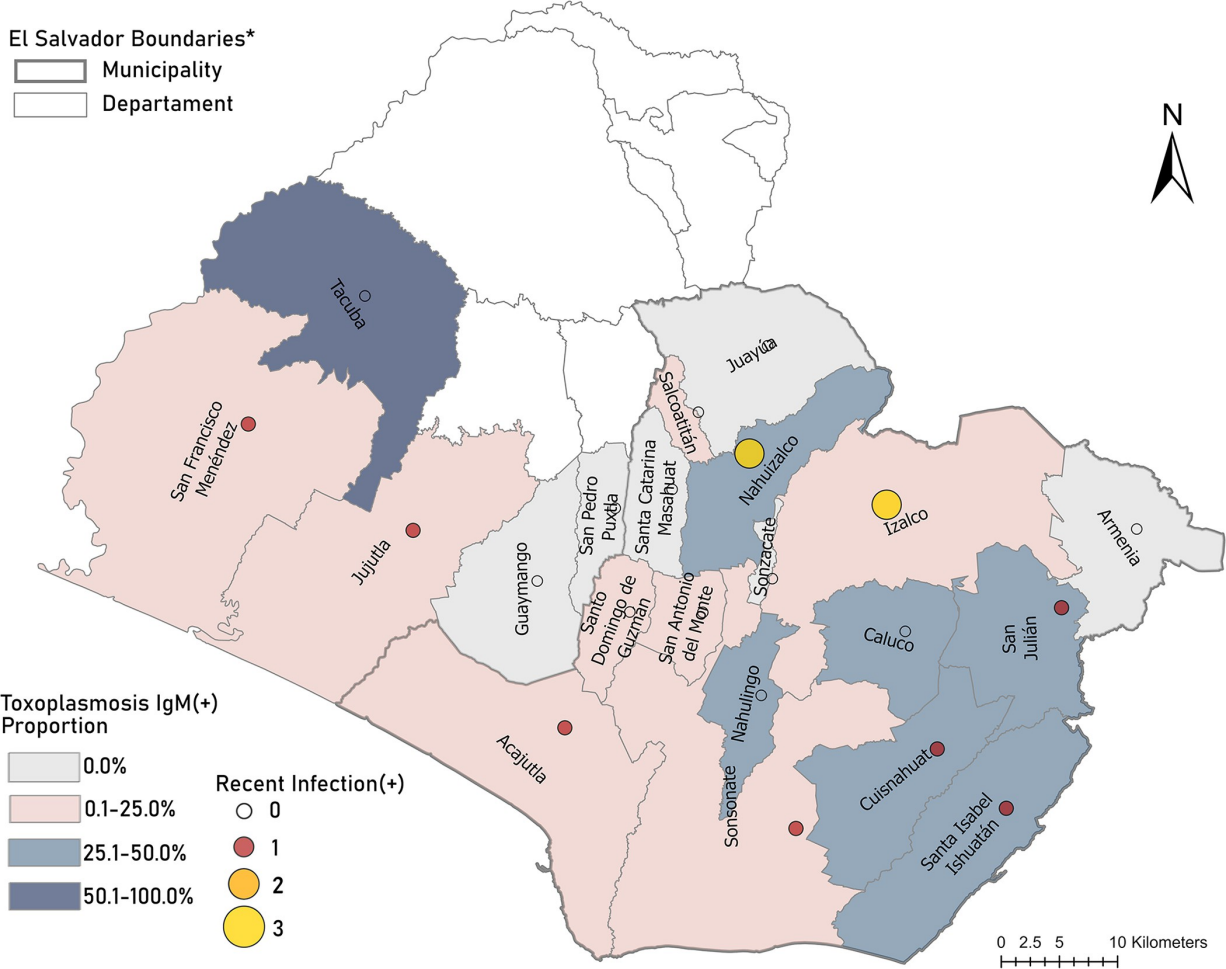
Cases of Salvadoran perinatal women with evidence of recent toxoplasmosis were primarily from two underserved municipalities in Sonsonate (Izalco, Nahuizalco). *ArcGIS map layers for El Salvador department and municipality boundaries are credited to Esri and Michael Bauer Research GmbH 2022, *Dirección General de Estadística y Censos*. https://www.esri.com/partners/michael-bauer-resear-a2T70000000TNZ3EAO.

This perinatal cohort demonstrated high historical toxoplasmosis burdens, with an overall 51.5% IgG seroprevalence (N = 102), and 20.2% IgM seroprevalence (N = 40). Among perinatal samples, 10.1% (N = 20) were seropositive for both IgG and IgM. The distribution of IgG seropositive case counts may be found in S1 Fig. Among all 198 perinatal samples, we identified two participants that meet classic diagnostic criteria for a recent *T*. *gondii* infection. One participant was IgG(+) and IgM(+) with low avidity and one participant IgG(+) and IgM(E) with low avidity. We found 31.1% (N = 59/190) of perinatal samples tested via dPCR were positive for *T*. *gondii* DNA, with 5.8% IgM(+) (N = 11/190) shown in Fig 2. In total, 6.6% of women (n = 13) had evidence of recent *T*. *gondii* infection within the last 16 weeks prior to childbirth, Fig 3. The method detection limit for the measured parasite DNA concentration was determined to be 0.01 copies/μL, which corresponds to < 1 positive partition per dPCR plate. Measured parasite DNA ranged from 0.04 to 0.56 copies per microliter. The detection of parasite DNA within these samples coupled with serologic results provides evidence of potential early infection among perinatal women. The majority of participants with evidence of recent toxoplasmosis infection were distributed throughout Sonsonate department, particularly Nahuizalco (N = 3) and Izalco (N = 3), shown in Fig 3.

Demographics and clinical history of the entire participant group can be found in Table 1. Among the entire cross-sectional perinatal group, median maternal age was 26 years (range: 15–45 years), and most resided in Sonsonate department. The majority of women reported overall good health status, though a small proportion had prior clinical history of miscarriage and pregnancy complications. Other relevant clinical histories present at the time of childbirth were gestational diabetes (N = 4), and history of seizure (N = 2). The majority of participants in this study lived in very low socioeconomic conditions with nearly 30% having significant substandard housing classified by barren earth floor, no electricity, or no potable water source in the home. Approximately 24% reported having pet cats and 15% of participants allowed animals to be inside the home, Table 1.

**Table 1:** Demographics and clinical profiles of Salvadoran perinatal women and results of Fisher’s exact test.

	Total Study Population (N = 198) % (N)	*T*. *gondii* (+) (N = 13) % (N)	*T*. *gondii* (-) (N = 185) % (N)	* P-value Fisher’s
Department				0.697
Ahuachapan	16.2% (32)	7.7% (1)	16.8% (31)	
Sonsonate	83.8% (166)	92.3% (12)	83.2% (154)	
Age Group				0.894
< 18 years	7.1% (14)	7.7% (1)	7.0% (13)	
18–25 years	43.9% (87)	53.8% (7)	43.2% (80)	
26–30 years	21.7% (43)	15.4% (2)	22.2% (41)	
31–45 years	27.3% (54)	23.1% (3)	27.6% (51)	
History of prior miscarriage				1.0
No	89.4% (177)	92.3% (12)	89.2% (165)	
Yes	10.6% (21)	7.7% (1)	10.8% (20)	
High Risk Pregnancy (current)				0.237
No	83.3% (165)	69.2% (9)	84.3% (156)	
Yes	16.7% (33)	30.8% (4)	15.7% (29)	
Prior pregnancy complications (any)				0.604
No	92.4% (183)	100% (13)	91.9% (170)	
Yes	7.6% (15)	0% (0)	8.1% (15)	
Chagas disease				0.181
Negative	93.9% (186)	84.6% (11)	94.6% (175)	
Positive	6.1% (12)	15.4% (2)	5.4% (10)	
Mother’s education level				0.544
< High school	69.2% (137)	61.5% (8)	69.7% (129)	
High School or greater	30.8% (61)	38.4% (5)	30.3% (56)	
Number of children				0.059
None	36.9% (73)	61.5% (8)	35.1% (65)	
1–3	59.6% (118)	30.8% (4)	61.6% (114)	
4+	3.5% (7)	7.7% (1)	3.2% (6)	
Significant substandard housing*				1.0
No	69.7% (138)	69.2% (9)	69.7% (129)	
Yes	29.8% (59)	30.8% (4)	29.7% (55)	
Total number in household				0.741
<3	20.2% (40)	23.1% (3)	20.0% (37)	
4–6	67.7% (134)	61.5% (8)	68.1% (126)	
7+	12.1% (24)	15.4% (2)	11.9% (22)	
Reported pet cats*				0.017
No	75.8% (150)	46.2% (6)	77.8% (144)	
Yes	24.2% (48)	53.8% (7)	22.2% (41)	
Animals inside the home*				1.0
No	85.4% (169)	84.6% (11)	83.8% (155)	
Yes	14.6% (29)	15.4% (2)	13.0% (24)	
Type of water*				0.131
None/natural water source	2.5% (5)	7.7% (1)	2.2% (4)	
Well	17.7% (35)	23.1% (3)	17.3% (32)	
Potable	79.3% (157)	69.2% (9)	80.0% (148)	

*some survey questions were left blank by participants therefore not all totals = 198; T. gondii recent (+) proportion out of N = 17 cases of suspected recent infection.

Clinical outcomes during the current pregnancy and neonatal birth outcomes are described in Table 2. Approximately 17% had current pregnancies considered high-risk and most women delivered via Cesarean section (c-section). More than 22% of this perinatal group had at least one complication during pregnancy and 10% of neonates had at least one negative health outcome at the time of birth. Labor and delivery complications included maternal vaginal infection at the time of birth, premature rupture of the membranes (PROM), perinatal asphyxia, and neonatal injury. More than 20% of neonates were admitted to the neonatal intensive care unit (NICU) for varying reasons, including acute respiratory distress syndrome (ARDS), low APGAR scores, premature birth, and low birthweight. Among perinatal women with evidence of recent toxoplasmosis infection, two had complications during pregnancy including threat of miscarriage and placenta previa. Four women in this group had complications at parturition including breeched birth and PROM. Neonatal birth outcomes among those with evidence of a recent infection included MAS and ARDS, shown in Table 2. Four infants among those with evidence of recent infection were admitted to the NICU.

**Table 2:** Current pregnancy and birth outcomes of Salvadoran perinatal women revealed significant associations between recent *T*. *gondii* infection and neonatal birth outcomes via results of Fisher’s exact test.

	Total Study Population (N = 198) % (N)	*T*. *gondii* (+) (N = 17) % (N)	*T*. *gondii* (-) (N = 181) % (N)	*P-value Fisher’s
Pregnancy Complications				
Any complications	22.2% (44)	15.4%(2)	22.7% (42)	0.737
Gestational Diabetes	2.0% (4)	7.7% (1)	1.6% (3)	0.240
Threat of miscarriage	9.6% (19)	23.1% (3)	8.6% (16)	0.115
Placenta previa or acreta	2.0% (4)	7.7% (1)	1.6% (3)	0.240
Cesarean section (c-section)	55.1% (109)	46.2% (6)	55.7% (103)	0.571
Breech birth	4.0% (8)	7.7% (1)	3.8% (7)	0.425
Urinary tract infection (UTI) during pregnancy	2.5% (5)	0% (0)	2.7% (5)	1.0
Labor and Delivery Complications				
Any	11.6% (23)	30.8% (4)	10.3% (19)	0.053
Premature rupture of membranes (PROM)	5.6% (11)	15.4% (2)	4.9% (9)	0.162
Neonatal Birth Outcomes				
Any symptom	10.1% (20)	23.1%(3)	9.2% (17)	0.137
Low birthweight	7.6% (15)	7.7% (1)	7.6% (14)	1.0
Apgar 1 min <9	7.1% (14)	7.7%(1)	7.0% (13)	1.0
Apgar 5 min <9	1.5% (3)	0% (0)	1.6% (3)	1.0
Admitted to neonatal intensive care (NICU)	20.2% (40)	30.8% (4)	19.5% (36)	0.476
Premature birth	5.6% (11)	0% (0)	5.9% (11)	1.0
Meconium aspiration syndrome (MAS)	2.5% (5)	15.4% (2)	1.6% (3)	0.037
Acute respiratory distress syndrome (ARDS)	5.6% (11)	7.7% (1)	5.4% (10)	0.543
Respiratory assistance (hood)	5.6% (11)	7.7% (1)	5.4% (10)	0.543

Fisher’s exact test revealed statistically significant univariate relationships between recent *T*. *gondii* infection and any labor and delivery complication (P = 0.053), shown in Table 2. Recent infection was also associated with neonatal MAS (P = 0.037) by Fisher’s exact test and multivariable logistic regression (OR:10.8, 95%CI:1.32, 80.0). Multivariable logistic regression revealed participants reporting pet cats had 4.94 times the probability of recent *T*. *gondii* infection (95%CI: 1.50, 17.14). Whereas, reporting increased wild animal species around the home was protective against recent infection (OR = 0.57, 95%CI: 0.31, 0.94).

## Discussion

This study found 6.6% of perinatal women from a vulnerable region of western El Salvador had evidence of recent *T*. *gondii* infection, defined in this study as infection within 16 weeks of parturition [24]. These 13 women’s neonates were significantly more likely to experience MAS, highlighting the impact of this perinatal infection. Results of this manuscript present evidence for the need to implement education campaigns and further serial screening studies of toxoplasmosis in pregnancy and congenital infection in Central America. According to Salvadoran and neighboring countries clinical guidelines for perinatal infection testing, HIV and syphilis tests are conducted on every woman receiving perinatal care; however, toxoplasmosis is only tested when women present symptomatically at the time of their perinatal care visit. As more than 80% of maternal infection present subclinically or with non-specific symptomology, our results provide evidence that primary or reactivated maternal infections may be going undiagnosed in highly vulnerable populations [27].

None of the neonates in this study had hallmark signs of congenital *T*. *gondii* infection at birth (hydrocephaly, intracranial calcifications, retinochoroiditis [9,27]); however, most congenitally infected neonates are asymptomatic at birth [9]. Clinical disease develops in approximately a quarter of initially asymptomatically infected neonates within the first year of life [28]. In the current study, neonates were clinically managed according to local medical guidelines, which do not require eye or neurological exam. In contrast, infected children born with clinically apparent disease are at an increased risk for severe long-term sequelae [28]. Within our study population, significant relationships between recent *T*. *gondii* infection and negative birth outcomes were noted, specifically MAS and complications at the time of birth. MAS has previously been associated with maternal infection or inflammatory response in pregnancy [29]. These findings warrant prospective evaluation as early identification and chemotherapeutic treatment are associated with improved long-term outcomes [30,31].

Statistical analysis revealed those mothers reporting pet cat ownership had nearly 5 more likely to show evidence of recent *T*. *gondii* infection. Domestic cats serve as a definitive host and an important contributor to human transmission, along with consumption of game meat and contaminated drinking water [32–34]. Transmission foci have further been previously reported in under-resourced populations, typically associated with environmental contamination clusters [32,35]. The current study found recent maternal *T*. *gondii* infection was associated with reporting fewer wildlife species seen around the home. This is likely an indicator of rural living where greater wildlife diversity is found. Given the distribution of cases combined with high multi-dimensional poverty in these two departments (e.g. high rates of crowding, and low access to potable water and sanitation services [36]), further environmental studies should evaluate the potential sylvatic sources leading to maternal infection locally. Additionally, higher case counts were observed in Nahuizalco and Izalco departments. Future studies should focus in these areas to investigate pet ownership, hand and food washing behaviors, as well as meat consumption behaviors among residents to better clarify the epidemiologic picture of toxoplasmosis in this region.

This pilot study identified 51.5% IgG seroprevalence among perinatal women in this study, consistent with previously estimates of IgG seroprevalence among pregnant women across El Salvador [10] and Latin American countries [37–39]. In contrast, this study group demonstrated a 10.1% IgM seroprevalence, a significantly higher rate than national estimates of 0.9% IgM perinatal seroprevalence, although the grey literature (e.g. university student theses) suggest that IgM seroprevalence varies greatly across the country [10,14,15]. Serologic methods for determining infection timing are difficult to interpret, as IgM can persist for months to years following recent infection and may further react with *T*. *gondii* antigens in the absence of veritable infection [11,12,40]. For this reason, we utilized both IgG avidity testing and dPCR to better assess acute infection in our study population. Toxoplasmosis avidity tests are typically performed when IgM and IgG serologic results are positive after repeated serial collections during the pregnancy [12]. *T*. *gondii* IgG avidity tests assess the strength of antigen-antibody binding, which may help to narrow down the timing of infection [11]—IgG avidity increases with time after exposure and changes from low binding to high binding approximately 6 months after primary infection [11]. In contrast, dPCR is an emerging technology for direct quantification of parasitic infections, with increased sensitivity and specificity for detection of low level-parasitemia [41,42]. Though *T*. *gondii* dPCR studies are limited, results have shown a six-fold higher positivity detection rate of *T*. *gondii* among a group of known positive meat samples and a 14- to 160- fold higher detection rate among banked clinical samples [25,42]. Conventional PCR has also been previously used to investigate acute infection in blood donors and pregnant women [26,43]. For this investigation of dPCR clinical utility potential, we conservatively classified those dPCR (+) and IgM (+) to avoid the potential for identification of latent infection using this highly sensitive molecular technique. As serologic results can be difficult to interpret, our findings support the utility of dPCR molecular methods in combination with serologic testing for *T*. *gondii* screening during pregnancy [12].

This study had a few limitations worth noting. Given the complexity of *T*. *gondii* diagnosis in the context of vertical transmission, serial samples are traditionally recommended; however, this study’s design was cross-sectional. To combat this concern, we applied conservative diagnostic profile requirements to define a positive case: 1) dPCR (direct detection of parasitic DNA) plus a positive IgM serologic result or a 2) positive antibody result with low avidity, criteria consistent with standard clinical guidelines. Due to the heightened sensitivity of dPCR compared to traditional and qPCR methods, it is further possible that detected parasite DNA represents exposure to the *T*. *gondii* protozoan in the absence of veritable clinical infection. Further validation studies of dPCR are necessary to determine the utility of this next generation molecular method in the clinical setting. Additionally, as a next-generation molecular technique, the application of dPCR may be costly. Therefore, the potential employment of this technique may be limited outside of reference laboratories in low resource settings. This study was also limited by the inability to capture some important infection risk factors, as the original survey was designed for a maternal Chagas disease surveillance study and participants not prospectively contacted for additional *T*. *gondii* transmission risk factor assessment. Lastly, we were unable to follow neonates to determine congenital infection in this study; however, the results of this study have been presented and shared with the hospital director and the Ministry of Health for follow-up care.

## Conclusion

Serology-based diagnosis of acute primary toxoplasmosis infection is complex, thus more effective and practical case diagnostics are critical in reducing the burden of congenital infections. This study provides supporting evidence that dPCR should be further explored for improved detection of acute *T*. *gondii* infection during pregnancy. Though cost may currently present limitations in lower resourced settings, future studies should be conducted to validate dPCR as a reliable infection testing method. The comparatively higher sensitivity of this method could aid in early detection of cases and reduce the need for multiple serologic tests in world regions that routinely screen for *T*. *gondii* throughout pregnancy. The current study identified high contemporary risk for undiagnosed congenital toxoplasmosis in the Western Region of El Salvador. The importance of this work highlights the need for education campaigns and enhanced future screening studies of pregnant women and their neonates in El Salvador and surrounding countries. Education and prenatal screening may present the opportunity for seronegative or immunocompromised pregnant women to avoid unnecessary exposure and for acutely infected women to obtain chemotherapeutics to prevent congenital transmission.

## Supporting information

S1 Table Targeted sequences used in digital PCR for molecular *Toxoplasma gondii* detection. (DOCX)

S1 Fig Case counts of toxoplasmosis IgG among perinatal women. Positive cases were highest in Acajutla, Izalco, and San Fransico Menendez municipalities in two departments in El Salvador with high multidimensional poverty. *ArcGIS map layers for El Salvador department and municipality boundaries are credited to Esri and Michael Bauer Research GmbH 2022, *Dirección General de Estadística y Censos*. https://www.esri.com/partners/michael-bauer-resear-a2T70000000TNZ3EAO.
(TIF)
