# Supplementary material for: Employing digital PCR for enhanced detection of perinatal Toxoplasma gondii infection: A cross-sectional surveillance and maternal-infant outcomes study in El Salvador
Source: PLoS Negl Trop Dis. 2024 May 20;18(5):e0012153. doi: 10.1371/journal.pntd.0012153 (PMC11142657; doi:10.1371/journal.pntd.0012153)
Supplement: S1 Table — (DOCX) [file pntd.0012153.s001.docx]

**S1 Table: Targeted sequences used in digital PCR for molecular *Toxoplasma gondii* detection.^1-3^**

| Sequence 5’-3’ | Oligonucleotide |
| --- | --- |
| GCT CCT CCA GCC CGT CCA AAC T | AF146527-Forward |
| TCC TCA CCC TCG CCT TCA T | AF146527-Reverse |
| FAM/MGB 59-AGG AGA GAT ATC AGG ACT GTA | Rep529 Taqman- Probe |

1. Gutierrez-Loli R, Ferradas C, Diestra A, Traianou A, Bowman N, Bok J, et al. Development of a novel protocol based on blood clot to improve the sensitivity of qPCR detection of Toxoplasma gondii in peripheral blood specimens. The American journal of tropical medicine and hygiene. 2019;100(1):83.

2. Homan W, Vercammen M, De Braekeleer J, Verschueren H. Identification of a 200-to 300-fold repetitive 529 bp DNA fragment in Toxoplasma gondii, and its use for diagnostic and quantitative PCR. International journal for parasitology. 2000;30(1):69-75.

3. Marino AMF, Giunta RP, Salvaggio A, Castello A, Alfonzetti T, Barbagallo A, et al. Toxoplasma gondii in edible fishes captured in the Mediterranean basin. Zoonoses and public health. 2019;66(7):826-34.
