# Supplementary figures and images for: Employing digital PCR for enhanced detection of perinatal Toxoplasma gondii infection: A cross-sectional surveillance and maternal-infant outcomes study in El Salvador
Source: PLoS Negl Trop Dis. 2024 May 20;18(5):e0012153. doi: 10.1371/journal.pntd.0012153 (PMC11142657; doi:10.1371/journal.pntd.0012153)

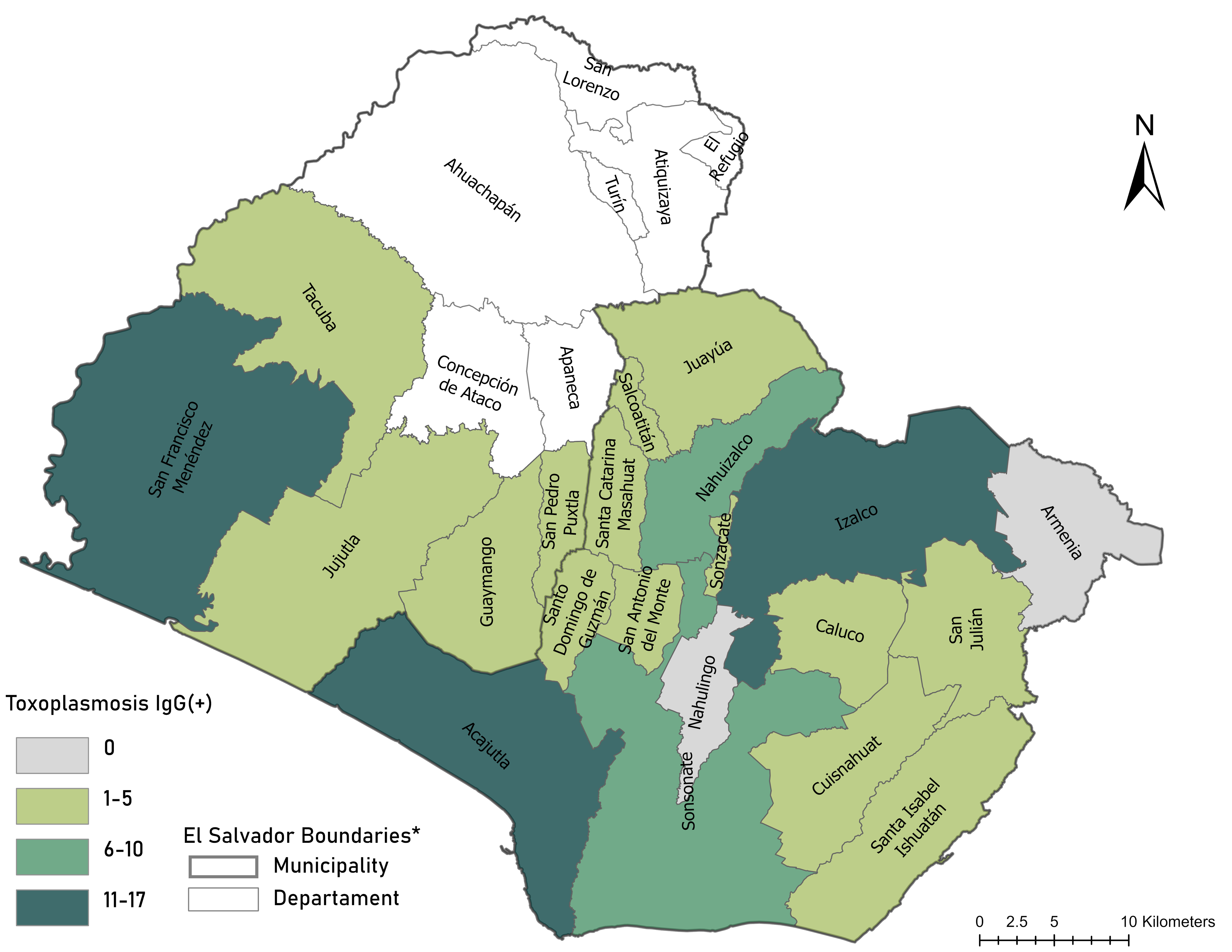

Supplement: S1 Fig — Positive cases were highest in Acajutla, Izalco, and San Fransico Menendez municipalities in two departments in El Salvador with high multidimensional poverty. *ArcGIS map layers for El Salvador department and municipality boundaries are credited to Esri and Michael Bauer Research GmbH 2022, Dirección General de Estadística y Censos. https://www.esri.com/partners/michael-bauer-resear-a2T70000000TNZ3EAO. (TIF) [file pntd.0012153.s002.tif]
